# Analysis of Interactions Occurring during the Pyrolysis of Lignocellulosic Biomass

**DOI:** 10.3390/molecules28020506

**Published:** 2023-01-04

**Authors:** Marcin Bielecki, Valentina Zubkova

**Affiliations:** The Institute of Chemistry, Jan Kochanowski University in Kielce, Uniwersytecka Str. 7, 25-406 Kielce, Poland

**Keywords:** interactions, lignin, cellulose, hemicellulose, pyrolysis

## Abstract

This paper presents a review of the recent advances in research on the interactions between the components of lignocellulosic biomass. The literature reports on the effects of interaction between lignocellulosic biomass components, such as cellulose–lignin, lignin–hemicellulose, and hemicellulose–cellulose, were discussed. The results obtained by other researchers were analyzed from the viewpoint of the interactions between the pyrolysis products formed along with the impact effects of the organic and inorganic components present or added to the biomass with regard to the yield and composition of the pyrolysis products. Disagreements about some statements were noted along with the lack of an unequivocal opinion about the directivity of interactions occurring during biomass pyrolysis. Based on the data in the scientific literature, it was suggested that the course of the pyrolysis process of biomass blends can be appropriately directed by changes in the ratio of basic biomass components or by additions of inorganic or organic substances.

## 1. Introduction

The overexploitation of fossil fuels and serious environmental problems connected with their excessive use have made authorities and scientists look for ‘clean’ and renewable alternative fuels [1,2,3]. Recently, a lot of attention has been paid to the use of biomass in the sustainable production of fuels and/or chemicals due to its wide availability and renewable and environmentally friendly nature [4,5,6]. Various thermochemical technologies including pyrolysis, gasification, combustion, hydrothermal liquefaction, and hydrothermal carbonization are used for the conversion of biomass to biochar, bio-oil, and non-condensing gases [7,8,9,10,11]. The biochar formed during biomass pyrolysis can be used as a fertilizer [12,13,14,15], a carbon dioxide absorber [16,17,18,19], a secondary fuel [20], a catalyst [21,22], or an adsorbent [23,24,25]. Additionally, some valuable chemical compounds are obtained from bio-oil [26,27]. Lastly, the gas formed during biomass pyrolysis can be re-used in the pyrolysis process as an agent that activates char [28] or changes the quality of the obtained bio-oil [29,30].

Hemicellulose, cellulose, and lignin are the basic components of lignocellulosic biomass. Moreover, there are small amounts of moisture and extracted material (including pectins) along with the inorganic components present in biomass [31,32,33,34,35]. More and more research papers devoted to the interaction between biomass components have recently appeared. Generally, the interaction between the basic components of the biomass is evaluated in model synthetic blends based on deviations from the rule of additivity which takes into account the pyrolytic behavior of single components [36,37,38,39,40]. The deviation from this rule is respected in the determination of the yields of char, tar, and volatile products [41,42,43,44,45,46], the composition of resultant products [36,37,47,48,49], or the structural-chemical parameters of the formed char [38,41,50]. The analysis of the results, conclusions, and suggestions presented in these works shows some inconsistencies that should be mentioned. 

This review aims to systemize the results obtained by different researchers on the interaction that takes place during the pyrolysis of lignocellulosic biomass. Such systematization will deepen the existing knowledge of the mechanism of pyrolysis of biomass and help to develop more reasonable processing methods.

## 2. Interactions between Basic Components of Biomass

### 2.1. Cellulose–Lignin Interactions

There are different opinions about the degree of interaction between simultaneously pyrolyzed biomass components such as cellulose and lignin. According to many authors, the interactions between cellulose and lignin should be considered strong [44,51,52,53,54]. However, Chang et al. [55] suggested that the interactions between these components are either apparent or do not occur at all. Guidicianni et al. [48] suggested that lignin promotes the cracking of cellulose. The presence of lignin affects the yield of obtained char but does not influence the composition of the volatile products. Fushimi et al. [56] thought that, on the one hand, lignin accelerates the volatilization of tar from cellulose and increases the yield of tar insoluble in water. On the other hand, the decomposition of lignin slows down as a result of its interaction with the char formed from cellulose. This reduces the yield of gaseous products and tar soluble in water. According to Worasuwannarak et al. [41], the yield of char increases, and the yield of tar decreases as a result of cellulose–lignin interactions during pyrolysis. These interactions are caused by the cross-link reactions between lignin and cellulose that occur with the formation of water vapor and compounds containing ester bonds. Zhang et al. [57] hold the view that the yields of pyrolysis products in a cellulose–lignin blend depend on the mass ratio of lignin and the temperature of pyrolysis. The influence of cellulose–lignin interactions on the yield of tar and gas is significant when the contribution of lignin exceeds 50%. In turn, Hosoya et al. [52] suggested that lignin exerts influence on cellulose and increases the formation of low molecular products from it. Wang et al. [58] thought that the cellulose–lignin interaction results in a decrease in the yield of char while cellulose breaks the decomposition of lignin and the formation of compounds containing a benzene ring in their structure. Hilbers et al. [59] focused on the influence of the crystallinity degree of cellulose on its interactions with lignin. In their opinion, the interaction between crystalline cellulose and lignin is stronger during slow pyrolysis. However, the presence of lignin does not affect the yield of products formed as a result of cellulose decomposition. They hold the view that cellulose–lignin interactions do not influence the yield of char, contrary to the opinions presented by other scientists [41,58]. Studying the behavior of cellulose and lignin in a native herbaceous biomass, Zhang et al. [55] stated that only apparent interactions occur. These authors did not note the occurrence of any interactions between cellulose and lignin in the case of wood biomass pyrolysis. 

Wu et al. [60] suggested that cellulose and lignin interact during the process of decomposition and thought that the low molecular weight products, namely, esters, aldehydes, acyclic ketones, and cyclic ketones, along with alcohols and phenols are formed in larger amounts during the co-pyrolysis of these two components. In their opinion, larger amounts of low molecular weight products are obtained as a result of the influence of lignin and compounds originating from its decomposition during the breakage of cellulose chains or the degradation of anhydrosugars and furans. However, Zhang et al. [57] proved that cellulose–lignin interactions increase the yield of char and facilitate the breakage of α-O-4 and β-O-4 bonds in the side chains of the lignin structure. According to Long et al. [61], the interactions in a binary blend of cellulose and lignin (1:1) resulted in a decrease in the mass loss of char. 

A general characterization of the effects connected with the influence of the cellulose–lignin interaction on the yields of pyrolysis products is presented in Table 1. 

It follows from Table 1 that scientists pay more attention to the research on the influence of lignin on the pyrolysis of cellulose. Attention should be drawn to the discrepancy between their opinions about the yields of pyrolysis products and the small number of publications devoted to lignin-cellulose interactions which makes it difficult to make more precise conclusions. 

### 2.2. Lignin–Hemicellulose Interactions

The data presented in the literature prove the lack of any indisputable opinion among scientists who study the interactions between hemicellulose and lignin. Hemicellulose includes mannan, xylan, and xyloglucan. However, xylan is the most abundant in nature, and it is a pre-dominant component of hemicellulose [62,63]. According to Geng et al. [64], the content of xylan can reach even 80% in some types of biomass. That is why in their works the scientists focus both on the interactions between the biomass components and hemicellulose and the interactions between the biomass components and xylan as a basic component of hemicellulose. Yu et al. [65] compared the pyrolytic behavior of single biomass components and their synthetic blends under simple and intimate mixing conditions. The results of their investigation do not prove the occurrence of any interactions in a xylan–lignin blend that led to the changes in the distribution of pyrolysis products. Usino et al. [66] noted small interactions in the decomposition of products during the pyrolysis of the xylan–lignin blend. In their opinion, the tar yield decreases as a result of these interactions, and the products originating from the decomposition of hemicellulose hinder the decomposition of most phenolic compounds. Despite this, the yield of 2-methoxyphenol and 4-vinyl-2-methoxyphenol increases. Zhou et al. [54] pointed out that the interaction between xylan and lignin does not influence the composition of formed gases. Moreover, these interactions cause a decrease in the concentration of polycyclic aromatic hydrocarbons present in tar originating from the xylan–lignin blend compared to their concentration in tar obtained from components that were pyrolyzed separately. Despite this, the interactions between xylan and lignin are not significant in their opinion [66].

In their turn, Hu et al. in [67] stated that there occurs a strong interaction between xylan and lignin during pyrolysis. In their opinion, this interaction leads to a decrease in the yield of formed char and the development of its porosity. Liu et al. [43] held the view that there is a strong interaction between hemicellulose and lignin. They thought that at T < 327 °C lignin increases the mass loss rate of hemicellulose but decreases the yield of 2-furaldehyde and compounds containing C = O group including aldehydes and ketones during the pyrolysis of hemicellulose. In turn, hemicellulose accelerates the pyrolysis of lignin and shifts this process towards lower temperatures but decreases the mass loss rate of lignin. To a great extent, hemicellulose promotes an increase in the yields of char and hydroxyacetaldehyde. The results of thermogravimetric investigations conducted by Wang et al. [58] also proved that the interactions between lignin and xylan cause lower mass losses during the pyrolysis of their blends. This can be connected with the fact that lignin hinders the formation of compounds containing carbonyl groups and C = C bonds that originate from the decomposition of xylan. A similar conclusion about the effects of the influence of lignin on xylan was drawn by Long et al. [61]. Furthermore, in their opinion, lignin restrains the decomposition of xylan. 

Kawamoto et al. [68] analyzed the influence of xylan and glucomannan on the pyrolysis of lignin. For this purpose, they used two model dimers of lignin of β-ether type, namely, [1-(4-hydroxy-3-methoxyphenyl)-2-(2-methoxyphenoxy)-1-propanol and 1-(3,4-dimethoxyphenyl)-2-(2-methoxyphenoxy)-1-propanol]. They suggested that the presence of xylan accelerates the pyrolysis of both phenolic and non-phenolic dimers. However, glucomannan shows a differential effect—it activates the pyrolysis of a phenolic dimer and hinders the pyrolysis of a non-phenolic one. 

Thus, the number of publications devoted to lignin–hemicellulose interactions is even lower than that related to cellulose–lignin interactions. Based on the aforementioned reports in the literature, it is impossible to make any unequivocal predictions about changes in the yield of the products originating from the blend of hemicellulose and lignin. 

### 2.3. Cellulose–Hemicellulose Interactions

The research conducted by Ding et al. [36] suggested that the interactions taking place between hemicellulose and cellulose during the pyrolysis process should be ignored. According to Hosoya et al. [52] and Long et al. [61], there is only a weak, insignificant interaction between hemicellulose and cellulose. Wang et al. [44] suggested that the influence of interaction between cellulose and hemicellulose is hardly possible to determine unequivocally. Nevertheless, in their opinion, the main reason for lower tar yields and higher yields in gas may be the influence of the interaction of these components on the transfer of heat and mass during heating. The pyrolyzed hemicellulose starts decomposing before cellulose and forms a liquid phase that can cover and wrap around the surface of the cellulose. This hinders the release of volatile products and facilitates the secondary degradation of the macromolecular compounds of cellulose. 

According to Wu et al. [51], the interaction between cellulose and hemicellulose facilitates the formation of low molecular weight compounds originating from the decomposition of hemicellulose. In this group of compounds, the authors include hydroxyacetone, acetone, acetic acid, 3-buten-2-ol, 1-hydroxy-2-butanone, and CO_2_. However, Usino et al. [66] claimed that the interaction between cellulose and xylan results in an increase in the number of formed aldehydes, especially hydroxyacetaldehydes, ketones, and furans. Wang et al. [69] suggested that the pyrolysis of hemicellulose causes the formation of many active centers that react easily with cellulose and hinder the pyrolysis of pure cellulose. This leads to the formation of a greater amount of low molecular weight compounds from hemicellulose. Liu et al. [43] suggested that at T > 327 °C, the changes in the DTG curve were caused by the influence of hemicellulose on cellulose; the mass loss rate decreases but the yield in char increases. Based on the results obtained by thermogravimetric investigations, Wang et al. [58] suggested that the interactions between cellulose and xylan shift the process of pyrolysis of a blend towards higher temperatures. Moreover, cellulose facilitates the formation of aromatic compounds that originate from the decomposition of xylan. A similar conclusion was made by Long et al. [61] in their research on the interactions of a binary blend of cellulose and xylan (1:1). According to Fushimi et al. [56], xylan accelerates the release of gases and tar soluble in water from cellulose. 

Yu et al. [65] compared the behavior of pyrolysis of single components with the behavior of their synthetic blends under simple and intimate mixing conditions. In their opinion, the interactions of cellulose and xylan cause an increase in char yield and a decrease in tar yield. These authors [65] suggested that natural biomass samples (oak, spruce, and pine) show a higher tar yield and a lower char yield compared to synthetic ones. The reason for the differences may be the porous structures of the studied samples. The presence of internal porosity and channels that facilitate the release of volatility plays a key role in the distribution of pyrolysis products. The lack of porosity in synthetic blends increases the possibility of the occurrence of re-polymerization reactions that lead to the formation of gas and char. 

**Figure 1:**
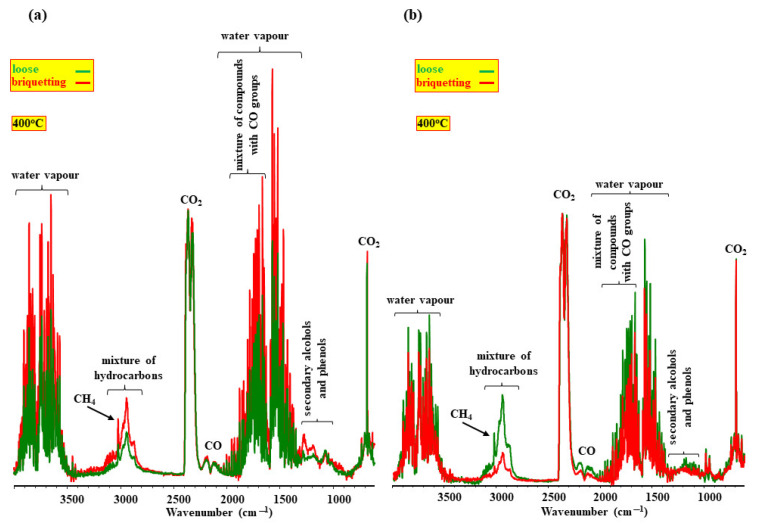
The FT-IR spectra of the volatile products of the pyrolysis of soft wood (**a**) and sunflower husks (**b**).

These suggestions on the influence of the porous structure of samples can be proved by research on loose and briquette samples [70]. The results of this research (Figure 1) show that briquetting changes the composition of the volatile products emitted during pyrolysis in a substantial way. Briquetting increases the contribution of saturated and unsaturated hydrocarbons in the composition of the volatile products of the pyrolysis of soft wood and decreases it in the volatile products of sunflower husks. This implies that it is not only the porosity of samples but also other factors that influence the yield of hydrocarbons during pyrolysis. 

**Figure 2:**
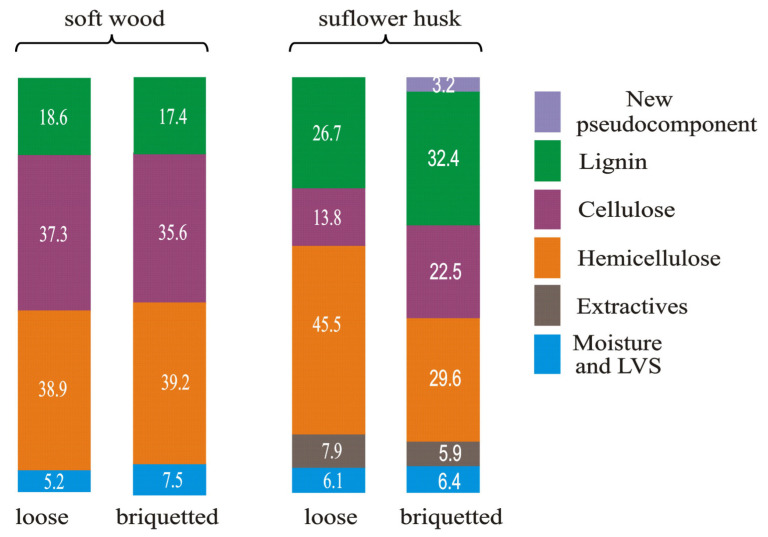
The scheme of changes in the contribution ratio of the biomass components of soft wood and sunflower husk samples that were caused by briquetting (based on data from [70]).

Figure 2 presents the scheme of changes in the contribution ratio of the biomass components of soft wood and sunflower husk samples that were caused by briquetting.

The biomass components were calculated on the basis of the deconvolution of the DTG curves [70]. This makes it possible to evaluate the contribution of every component in the formation of these curves. Indeed, the deconvolution results reflect the changes in the thermal stability of the biomass components. In the case of sunflower husk samples, briquetting increases the contribution of lignin and cellulose in the formation of the DTG curve (i.e., lowers their thermal stability) and decreases the contribution of hemicellulose (i.e., increases its thermal stability). This leads to a decrease in the contribution of hydrocarbons in the composition of the volatile products of pyrolysis. In the case of soft wood samples, the differences in the contribution ratio of biomass components in the formation of DTG curves of loose and briquette samples are much smaller. However, for the briquette soft wood sample, a decrease in porosity increases the content of hydrocarbons in the composition of the volatile products of pyrolysis.

A general characterization of the influence of interactions occurring in the blend of hemicellulose (alternatively, xylan) and cellulose on the yield of pyrolysis products is presented in Table 2. 

The data in Table 2 imply that the yield of char and volatile products increases during the interaction in the blend of hemicellulose and cellulose irrespective of the type of acting component. 

**Figure 3:**
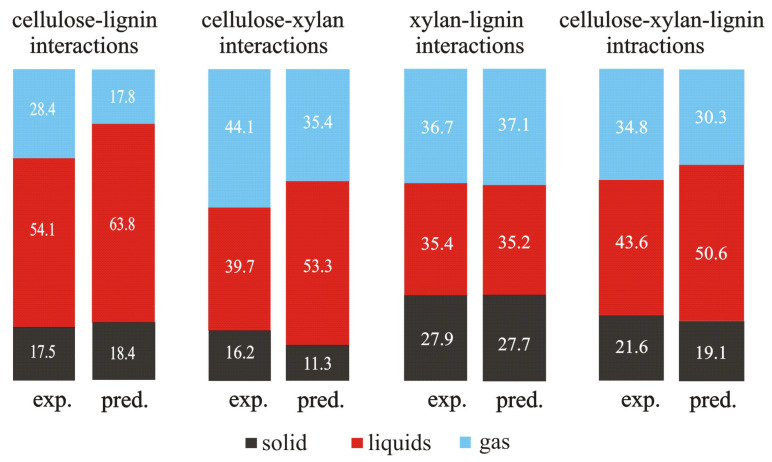
The experimentally measured and predicted yields of solid, liquid, and gaseous products at the pyrolysis temperature of 520 °C (based on the data from [65]).

Based on the data taken from [65], Figure 3 presents a comparison between the values of the experimentally measured yields of the solid, liquid, and gaseous products of the blends of single components and the predicted values of yields calculated according to the additivity rule. 

The differences in the measured and predicted values for the two binary blends (cellulose–lignin and cellulose–xylan) and the three-component one (cellulose–xylan–lignin) are observed. This fact points out the occurrence of interactions between the components in these blends. The only exception is the binary blend of xylan and lignin. 

**Figure 4:**
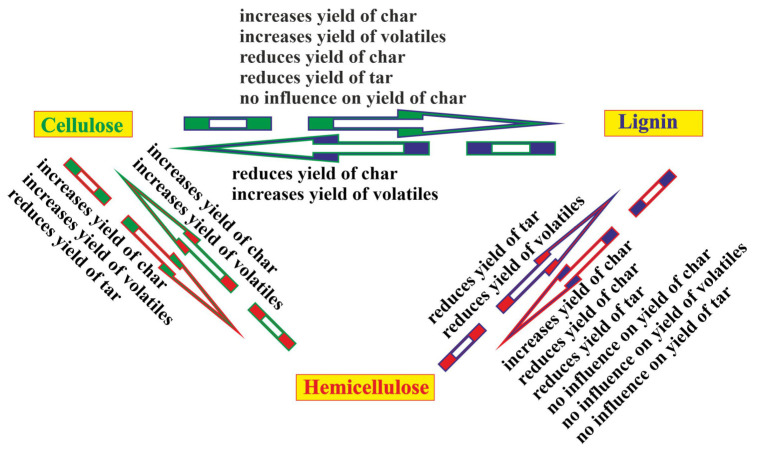
The effects of the interactions between the basic biomass components: cellulose increases yield of char (data from [41,48,57]); increases yield of volatiles (data from [52,60,67]); reduces yield of char (data from [52]); reduces yield of tar (data from [41,56,57]); no influence on yield of char (data from [55,59]); lignin reduces yield of char (data from [58]); increases yield of volatiles (data from [56]); hemicellulose reduces yield of tar (data from [52]); reduces yield of volatiles (data from [43]); lignin increases yield of char (data from [43]; reduces yield of char (data from [67]); reduces yield of tar (data from [58]); no influence on yield of char (data from [65]); no influence on yield of volatiles (data from [65]); no influence on yield of tar (data from [65]); hemicellulose increases yield of char (data from [43]); increases yield of volatiles (data from [51]); cellulose increases yield of char (data from [43]); increases yield of volatiles (data from [44,56,66]); reduces yield of tar (data from [44,65]).

The schematic relationship of the interactions between the basic biomass components discussed above is well reflected in Figure 4. 

The schema in Figure 2 implies that the effects of the direct interactions between cellulose and lignin, lignin and hemicellulose, and hemicellulose and cellulose influence the course of the pyrolysis process of the blend of all three components and native biomass in an indirect way. Therefore, it is impossible to predict the pyrolytic behavior of a three-component blend on the basis of the pyrolytic behavior of single components. Despite some ambiguity in the conclusions made by the scientists, it can be suggested that the selection of different biomass types for the composition of a blend changes the yield and composition of the pyrolysis products. In this case, the total content of basic biomass components in a blend should be taken into account.

## 3. Interactions between Products Formed during Pyrolysis Process

During the heating of the biomass, interactions can take place not only between the biomass basic components but also between the products of their pyrolysis being formed. The authors of works [71,72] analyzed the interactions between the volatile products of the pyrolysis of biomass components. It follows from the results obtained by Chen et al. [71] that the yield of volatile products increases and the yield of liquid products decreases during the interactions between the volatile products of the pyrolysis of unmixed biomass components. The interaction between compounds in volatile products facilitates the decomposition of heavier molecules into lighter ones. This leads to a decrease in the amount of formed liquid products. The authors [71] suggested that the interaction between volatile compounds originating from hemicellulose and lignin is much greater than that between volatile compounds from cellulose and hemicellulose and cellulose and lignin. 

Hosoya et al. in [72] present the results of research on the interactions between volatile products originating from cellulose and the formation of tar and char from lignin as well as between solid and liquid products. In this research, they also focus on the influence of products originating from lignin on cellulose. In their opinion, the interaction results in an increased yield of tar from cellulose and lignin, an increased yield of gaseous products, and a decreased yield of char from cellulose and lignin. The scientists in [72] stated that a variable impact on the yield of liquid products was caused by the influence of volatiles from cellulose on volatiles from lignin. Namely, the yield of liquid products from cellulose decreases, and that of liquid products from lignin increases. Moreover, the authors of [72] suggested that pyrolysis products originating from cellulose act as hydrogen donors, and radicals formed from lignin act as their acceptors. 

Information on the existence of interactions between the volatile products of pyrolysis and the formed char can be found in other publications [38,73,74,75,76,77]. Yang et al. [38] analyzed the secondary reactions between the volatile and solid products of cellulose, hemicellulose, and lignin. In their opinion, the interaction between the volatiles from cellulose and hemicellulose and the solid products of pyrolysis of a three-component blend results in a decrease in the yield of gaseous products and an increase in the yield of liquid products, whereas in the case of volatile compounds from lignin the reverse effect is observed after interaction of the blend with char. 

In their turn, investigating the influence of char on the decomposition of tar during the pyrolysis of rice straw, Song et al. [73] noted a decrease in tar yield as a result of the interaction between the volatile compounds and char. The scientists thought that the functional groups containing oxygen on the surface of char are responsible for the destruction of formed tar. In their opinion, rinsing the biomass with water and acid facilitates the decomposition of tar during the interaction between volatiles and char. Continuing their research on the mechanism of interactions between volatiles and char, Song et al. [74] emphasized the importance of water vapor during the pyrolysis of rice straw. They state that, under the influence of water vapor, the interactions between the volatile compounds and char can substantially decrease the yield of compounds with aromatic ring systems in the products of pyrolysis of rice straw. As a result of the interaction with water vapor, oxygen-containing functional groups are formed only on the surface of the char, which causes the destruction of tar during the interaction between volatiles and char. According to Gao et al. [75], the interactions between the volatile products and char (at the same time) during the pyrolysis of poplar wood cause an increase in yield of both solid (i.e., char and ash) and gaseous products and a decrease in the yield of liquid products. The structure of the pores in char plays a very important role in this interaction. 

In their investigations, the authors of [76,77] focused on the influence of selected compounds present in the composition of volatile products on the interaction with char. Huang et al. [76] stated that under the influence of benzyl phenyl ether, the activity of char gradually decreases with an increase in temperature. In their opinion, the reason lies in the elimination of groups containing oxygen on the surface of char at higher temperatures. In turn, Ding et al. [77] analyzed the interaction between char and (benzyloxy)benzene. They proved that char from xylan decomposes (benzyloxy)benzene more actively than char from lignin and cellulose. The significant differences in their activity result mainly from the presence of C(sp^2^)-O groups and C(sp^3^)-O groups on the surface of the char. Smith et al. [78] and Chen et al. [79] reported that hydrogen bonds between compounds with groups containing oxygen are formed on the surface of char as a result of the interaction between char and volatiles. According to Huang et al. [80] and Liu et al. [81], functional groups containing oxygen play a key role in the interactions between volatiles and char. Due to the complexity of interactions between volatiles and char during biomass pyrolysis, Huang et al. [80] allowed some simplifications in their work. The scientists used a β-5 lignin dimer as a model compound of a lignin-derived volatile whereas the model substances of char were a graphitized multi-walled carbon nanotube (CNT) and an amino-modified CNT. The experiment showed a greater conversion of the model substance in the presence of amino-CNT. An increase in the conversion of the model compound was explained by the interactions of the amino-hydroxyl groups and a greater electronegativity of oxygen in the hydroxyl group that occurred as a result of the breakage of the C-O bond in the dimer. In turn, Li et al. [81] conducted model research on the interactions between char and volatiles that contained feeding gas, bio-oil (tar), and steam in their composition. They stated that the activation of char by water vapor during pyrolysis leads to an increase in C-O-containing functional groups (including aromatic C-O groups) in this char. Such activation causes a decrease in the contribution of tar in pyrolysis products. However, Xiong et al. [82] reported that the interactions between volatiles and char depend on the heating rate. At lower heating rates, these interactions increase the yield of macromolecular compounds in bio-oil whereas, at high heating rates, they increase the number of formed lipids. 

It follows that in the aforementioned investigations the scientists draw different conclusions with regard to the yields of liquid and gaseous products formed during the interactions between volatiles and char both for a blend of single components and native biomass. The number of reports in the literature devoted to interactions of this type is not sufficient enough to draw definite conclusions from these observations. It also cannot be excluded that the discussed interactions are influenced by factors other than those presented in the aforementioned publications.

## 4. The Influence of Interaction between Components on the Formation of Levoglucosan

Levoglucosan (1,6-anhydro-β-d-glucopyranose) is a widely known indicator of biomass burning and that pyrolysis is the initial stage of the combustion process [83,84,85]. During pyrolysis, volatile, liquid, and solid products are formed that are burned in the next stage [86]. The interactions between biomass components during pyrolysis change the yield and composition of products and thus can influence the formation of levoglucosan. Due to this, the influence of the aforementioned interactions on its formation during pyrolysis as the first stage of the combustion process gains meaning. According to many authors, the formation of levoglucosan is affected by the interactions between biomass components, i.e., between cellulose, hemicellulose, and lignin [38,56], between cellulose and lignin [42,44,52,53,55,59,72,87], and between cellulose and hemicellulose [42,43,44,51,52]. 

The authors of [38,56] stated that the interaction of cellulose, hemicellulose, and lignin causes a decrease in the yield of levoglucosan. Yang et al. [38] reported that the amount of formed levoglucosan decreases as a result of the interactions between volatile compounds of cellulose and solid products obtained from hemicellulose and lignin. In their opinion, ash from hemicellulose and char from lignin catalyze the decomposition of levoglucosan. Fushimi et al. [56] had a similar opinion about the effect of interaction between the three biomass components. On the basis of an HPLC analysis of tar obtained from a blend of cellulose, xylan, and lignin, they noted a decrease in the yield of levoglucosan. In their opinion, the presence of lignin and xylan made the released volatiles of levoglucosan decompose into non-condensable gases. They suggested that levoglucosan can polymerize into char. Kawamoto et al. [42] showed the results of the separate pyrolysis of levoglucosan, cellulose, lignin, and xylan. They stated that the volatile compounds originating from the pyrolysis of cellulose and lignin promoted the decomposition of levoglucosan whereas the volatiles from xylan restrained it. 

The yield of levoglucosan can be influenced by the interactions between cellulose and lignin [53,55,72,87]. According to Zhang et al. [55], lignin favors the formation of furans and low molecular weight compounds during the breakage of glycosidic bonds in cellulose. Many authors [53,68,79] thought that a decrease in the yield of levoglucosan can be connected with the combination of radicals formed during the decomposition of lignin and the formation of hydrogen bonds on the surface of lignin [87]. During the decomposition of levoglucosan, non-condensable gaseous products [72] and low molecular weight compounds such as phenols, guaiacols, and syringols are formed [53]. 

Opposed to this opinion, the authors of works [44,52,59] stated that the interactions between lignin and cellulose increase the number of formed anhydrosugars, especially levoglucosan. Wang et al. [44] justified their views by the measurements of changes in the surface of peaks in the GC-MS spectra of liquid products. Hosoya et al. [52] proved their opinion with the data of a GC-FID analysis of tar composition. According to Hilberts et al. [59], an increase in the yield of levoglucosan is caused by the contribution of lignin in the inhibition of its dehydration reaction. 

Investigations connected with the interactions between cellulose and hemicellulose are no less important. According to the authors of works [43,44,51,52], the interactions between cellulose and hemicellulose reduced the formation of products originating from the decomposition of cellulose, especially the formation of levoglucosan. Liu et al. [43] held the view that, when mixed with cellulose, hemicellulose restrains the release of volatiles from cellulose. As a result of the secondary reactions of volatiles from hemicellulose with volatiles from cellulose, levoglucosan transforms into hydroxyacetaldehyde and other low molecular weight volatile compounds. On the basis of the analysis of changes on the surface of peaks in GC-MS and GC spectra of liquid products, Wang et al. [44] stated that the interaction between cellulose and hemicellulose restrains the formation of levoglucosan. Wu et al. [51] concluded that the interactions between cellulose and hemicellulose promoted the formation of low molecular weight compounds originating from hemicellulose at the expense of inhibition of the formation of the products from cellulose decomposition, especially levoglucosan. In turn, based on the analysis of a fraction of tar soluble in isopropanol, Hosoya et al. [52] stated that the yield of levoglucosan decreased as a result of the interactions between cellulose and hemicellulose in the form of xylan and glucomannan. In their opinion, this is connected with the melting of hemicellulose to liquid form during heating. During pyrolysis, a layer of molten hemicellulose covers cellulose. That is why the formation of volatile products from cellulose is restrained whereas the release of products originating from the decomposition of hemicellulose is favored. 

A systematization of the aforementioned research results of the influence of the interactions between biomass components on the yield of levoglucosan during pyrolysis is presented in Table 3. 

It follows from Table 3 that a lot of publications devoted to the yield of levoglucosan focused on the interactions between cellulose and lignin. However, the conclusions on the effect of the influence of these interactions made by the authors are contradictory to each other. Attention should be drawn to the fact that the presence of hemicellulose in studied samples can negatively affect the yield of levoglucosan. This implies that the yield of levoglucosan in the volatile products of pyrolysis can be controlled by the addition of hemicellulose or its components to the pyrolyzed biomass. Such a suggestion stands in need of extensive research focused on the explanation of the mechanism of influence exerted by hemicellulose on the yield of levoglucosan.

## 5. The Influence of Inorganic Components on the Course of Biomass Pyrolysis Process

Apart from basic components, the presence of inorganic components was ascertained in plant biomass [88,89,90]. These inorganic components can affect the changes in yield and composition of pyrolysis products. 

### 5.1. Inorganic Components and the Yield of Pyrolysis Products 

According to many authors [49,91,92,93,94,95,96,97], inorganic components influence the yield of products of native biomass pyrolysis. For example, Gargiulo et al. [49] stated that during pyrolysis, Na and K ions present in *Arundo donax* decrease the yield of liquid products and char and increase the yield of gaseous products. Eom et al. [91] impregnated poplar wood samples with solutions of potassium, calcium, and magnesium chlorides. They suggested that the presence of KCl during biomass pyrolysis decreases the yield of volatile compounds and increases the yield in char. On the contrary, the presence of CaCl_2_ increases the yield of gases and decreases the yield of solid products. In turn, MgCl_2_ does not show any constant activity. At lower concentrations, MgCl_2_ decreases the yield of volatile compounds but increases the yield of char. At higher concentrations, this compound does not influence the yields of particular products. After impregnating a poplar wood sample with KCl solution, Hwang et al. [92] stated that the presence of K lowers the maximum temperature of the biomass composition, increases the yield of tar, and decreases its viscosity. After covering rice husks with MgO, MgCO_3_, CaO, and CaCO_3_, Shen et al. [93] reported that under the conditions of flash pyrolysis, such a procedure facilitates a decrease in the yield of bio-oil and increases the yield of char and gaseous products. Hu et al. [94] analyzed the influence of alkali and alkaline earth metals (AAEM) present in rice husks on the course of their flash pyrolysis. They compared the obtained results with those for the samples of rice husks demineralized in HCl. This gave them grounds to state that the presence of AAEM in rice husks increases tar yield and decreases char yield. In their research, Guo et al. [95] impregnated pine wood samples with a KNO_3_ solution. In their opinion, the yield of char increases with a rise in K concentration; the yield of tar lowers, and the yield of pyrolytic gases grows proportionally. 

Collard et al. [96] impregnated beech wood samples with Fe(NO_3_)_3_ and Ni(NO_3_)_2_ solutions. They stated that the presence of both metals caused an increase in the yield of char and gaseous products and decreases the yield of tar. A similar opinion was proposed by Xia et al. [97] after they investigated the samples of Chinese chestnut shells impregnated with a Fe(NO_3_)_3_ solution. The researchers thought that the presence of Fe increases the yield of gases and char and decreases the yield of tar.

It was noticed that inorganics influence the thermochemical changes of single biomass components [96,98,99,100]. In this regard, attention should be drawn to the work by Collard et al. [96] that was devoted to the impregnation of biomass components, i.e., cellulose, xylan, and lignin, with Fe(NO_3_)_3_ and Ni(NO_3_)_2_ solutions. Similar to Chinese chestnut shells, during the pyrolysis of cellulose, these compounds caused an increase in the yield of char and gases and a decrease in the yield of tar. During the pyrolysis of lignin, these compounds increased the yield of char and decreased the yield of tar. Moreover, scientists in [96] noted that the interactions of Fe with xylan caused an increase in the yield of tar and gases but did not affect the yield of char. Ni interacts with xylan, otherwise reducing the yields of tar and char and increasing the yields of volatile products. 

Khelfa et al. [98] impregnated biomass components with MgCl_2_ and NiCl_2_ solutions. They stated that, during the pyrolysis of cellulose, MgCl_2_ substantially increased the yield in char whereas during the pyrolysis of xylan and lignin both salts did not affect the yield of char. Trubetskaya et al. [99] impregnated lignin with KNO_3_. They reported that the decomposition of lignin K decreases the yield of tar and soot. Fan et al. [100] covered cellulose, xylan, and lignin with KCl. They suggested that such impregnation increases the yield of char during the pyrolysis of cellulose and decreases it during the pyrolysis of lignin. In the case of the pyrolysis of xylan, the impregnation with KCl increased the yield of volatile products. 

**Figure 5:**
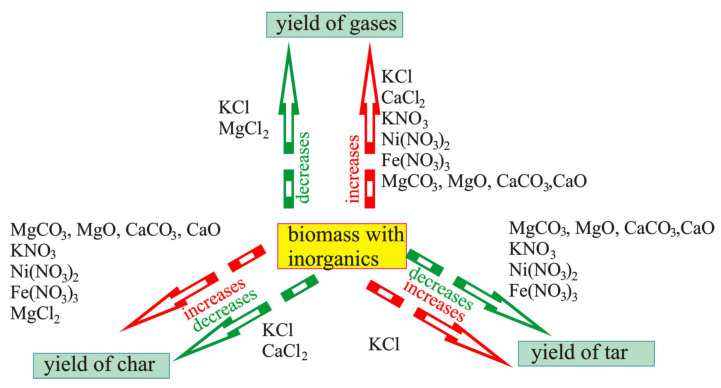
The influence of impregnation on the yields of particular pyrolysis products: increase in gas yield influenced by KCl (data from [101]); CaCl_2_ (data from [90]); KNO_3_ (data from [99]); Ni(NO_3_)_2_ (data from [91]); Fe(NO_3_)_3_ (data from [91,98]); MgCO_3_, MgO, CaCO_3_, CaO (data from [97]); decrease in gas yield influenced by KCl and MgCl_2_ (data from [90]); increase in char yield influenced by MgCO_3_, MgO, CaCO_3_, and CaO ( data from [97]); KNO_3_ (data from [99]); Ni(NO_3_)_2_ (data from [91]); Fe(NO_3_)_3_ (data from [91,98]); MgCl_2_ (data from [90,100]); decrease in char yield influenced by KCl (data from [101]) and CaCl_2_ (data from [90]); increase in tar yield influenced by KCl (data from [96]); decrease in tar yield influenced by MgCO_3_, MgO, CaCO_3_, and CaO (data from [97]); KNO_3_ (data from [99,102]); Ni(NO_3_)_2_ (data from [91]); Fe(NO_3_)_3_ (data from [91,98]).

Summarizing all the above, it should be underlined that inorganic components both present in biomass and added during impregnation cause ambiguity in statements made by different authors. Figure 5 generalizes the influence of inorganic components used during impregnation on the yield of pyrolysis products.

It follows from Figure 5 that impregnation with KCl can facilitate an increase in the yield of gases [100], char [91], and tar [92] or lower the number of gases [91] and char [100]. Iron, nickel, and potassium nitrates affect an increase in the yield of char and a decrease in the yield of tar [95,96,97] from selected biomass components and their blend in a more or less unambiguous way. 

### 5.2. The Influence of Inorganic Components on the Composition of Volatile Products of Pyrolysis 

The information mentioned above implies that during the thermochemical processing of biomass, inorganic components both naturally present or added can play an important role. That is why investigations connected with the influence of inorganics on the changes in the composition of volatile products of native biomass pyrolysis [94,95,97,102] and single components of biomass [48,49,99,100,101] occupy a special place in the scientific literature. 

**Figure 6:**
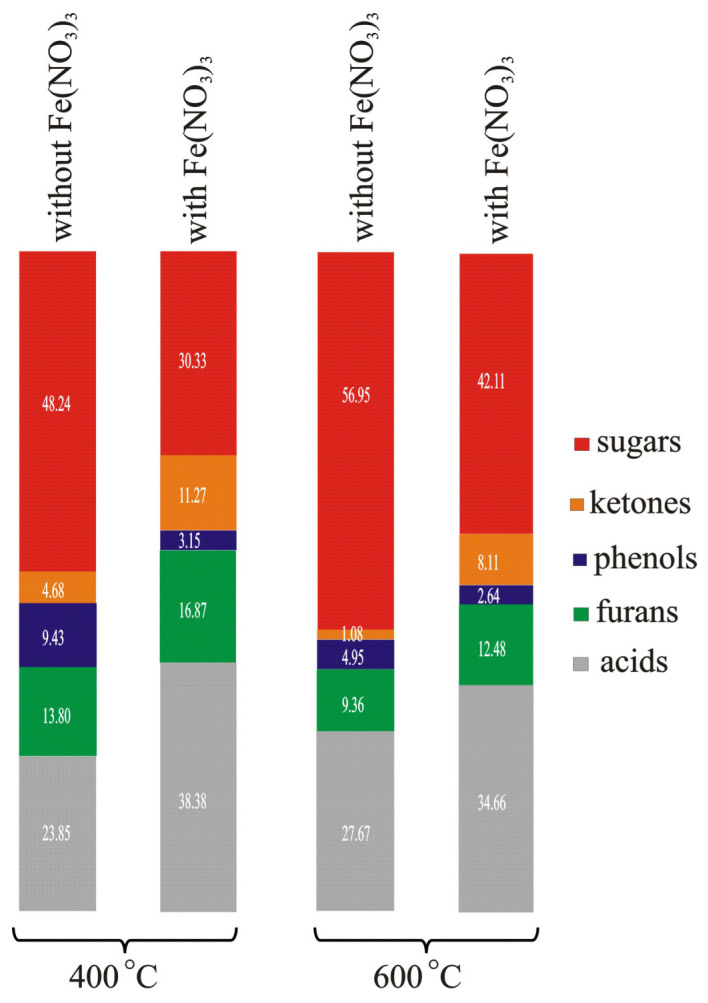
The changes in the contents of sugars, ketones, phenols, furans, and acids in the volatile products of the pyrolysis of Chinese chestnut shells caused by an increase in temperature from 400 °C to 600 °C and the addition of Fe(NO_3_)_3_ (based on data from [89]).

Hu et al. in [94] presented the results of research on the influence of AAEM present in rice husks on the composition of gaseous products. The authors showed that the influence of AAEM on the pyrolysis of these samples causes a decrease in the yield of CO_2_, and an increase in the yield of CO, H_2_, and C_2_H_4_. In their investigation, Chen et al. [102] covered cotton stalk samples with CaO. In their opinion, CaO can act as an absorbent, a reagent, and a catalyst, depending on the pyrolysis conditions used. Acting as an absorbent, it causes a decrease in the content of CO_2_ in gaseous products. Acting as a reagent, it causes an increase in the content of ketones in tar and a decrease in the content of acids and phenols. Acting as a catalyst, it increases the content of furans and hydrocarbons and decreases that of esters and anhydrosugars. After impregnating the samples of Chinese chestnut shells with a Fe(NO_3_)_3_ solution, Xia et al. [97] ascertained an increase in the content of H_2_ and CO_2_ and a decrease in CH_4_ in gaseous products with the rise of pyrolysis temperature. The data taken from [89] imply that with an increase in temperature, the contents of sugars, ketones, phenols, furans, and acids in the composition of volatile products of chestnut shells change under the influence of the addition of Fe(NO_3_)_3_ (Figure 6).

Impregnating pine wood with KNO_3_ solution, Guo et al. [95] stated that under the influence of K, the yield of gases H_2_ and CO_2_ increases but that of CO and CH_4_ decreases.

Giudicianni et al. [48] analyzed the influence of AAEM present in hemicellulose. They pointed out that the presence of AAEM influences the mechanism, yield, and properties of products of the pyrolysis of hemicellulose. These compounds caused a decrease in the yield of CO_2_ and an increase in the yield of CO and H_2_. Gargiulo et al. [49] impregnated cellulose with NaCl and KCl solutions. They reported that during the pyrolysis of cellulose, the inorganic components containing Na and K atoms increased the yield of CO_2_ and H_2_ in the composition of volatiles at the expense of decreasing the yield of CO. Chen et al. [101] coated cellulose, hemicellulose, and lignin with CaO powder. They determined that during pyrolysis at a temperature below 600 °C, the interaction of cellulose, hemicellulose, and lignin with CaO decreased the yield of formed CO_2_ in volatile products. CaO did not change the yield of CH_4_ during the pyrolysis of cellulose, it lowered the yield of CH_4_ during the pyrolysis of hemicellulose and increased the yield of CH_4_ during the pyrolysis of lignin. In their research, Trubetskaya et al. [99] impregnated lignin with a KNO_3_ solution. They demonstrated that the impregnation lowers the number of light hydrocarbons and CO but raises the amount of CO_2_ in volatile products originating from the decomposition of lignin. Covering cellulose, xylan, and lignin with KCl solution, Fan et al. [100] obtained a greater yield of CO_2_ in the composition of volatiles. In their opinion, potassium favors the cleavage and reformation of carboxyl (-COOH) and carbonyl (>C = O) groups. 

Contradictions presented in opinions expressed by different authors concern the influence of inorganics on the yield of levoglucosan. For example, according to the authors of the works [49,91,103,104,105], the interactions of Na, K, Ca, and Mg atoms with biomass components lowered the yield of levoglucosan whereas Ni atoms increased it [96]. The investigations of the influence of inorganics on the formation of levoglucosan include the pyrolysis of native biomass [91,94,96] as well as the pyrolysis of cellulose [49,95,96] as main providers of this compound.

For example, Eom et al. [91] impregnated poplar wood samples with potassium, calcium, and magnesium chlorides. In their opinion, Ca and K restrain the formation of levoglucosan and lower the yield of furans and pyrans but increase the yield of low molecular weight compounds such as glycolaldehyde, acetic acid, acetol, and butanediol. Hu et al. [86] investigated the influence of K, Na, Ca, and Mg present in rice husks on the yield of formed levoglucosan. They determined that Ca and Mg affect the decomposition of levoglucosan more substantially than K and Na. Collard et al. [96] impregnated a beech wood sample with a Ni(NO_3_)_2_ solution. They ascertained an increase in the production of levoglucosan in the wood with a low Ni content.

**Figure 7:**
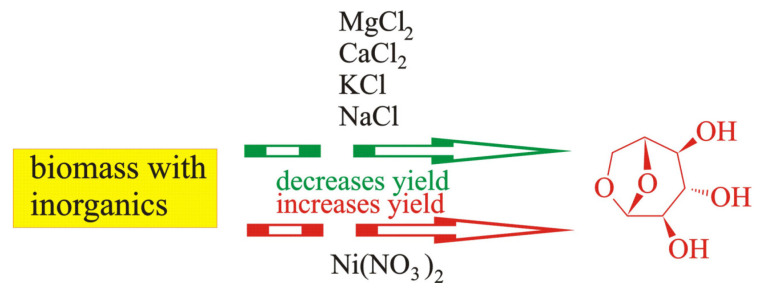
The influence of impregnation on the yield of levoglucosan: decrease in yield of levoglucosan influenced by MgCO_3_, MgO, CaCO_3_, and CaO (data from[97]) ; KNO_3_ (data from [99,102]); Ni(NO_3_)_2_ (data from [91]); Fe(NO_3_)_3_ (data from [91,98]); increase in yield of levoglucosan influenced by Ni(NO_3_)_2_ (data from [96]).

Gargiulo et al. [49] impregnated cellulose with NaCl and KCl solutions. They determined that the yields of levoglucosan and furans were lower in the presence of Na and K ions, whereas the influence of Na ions was more efficient than that of K ions. Kawamato et al. [103] as well as Shimada et al. [104] covered cellulose with solutions of sodium, potassium, magnesium, and calcium chlorides. Kawamato et al. [103] additionally used LiCl. They stated that the introduction of Na, K, Ca, and Mg atoms into cellulose caused a decrease in the yield of levoglucosan. The aforementioned is schematically presented in Figure 7.

It follows from Figure 7 that most inorganic components (KCl, NaCl, MgCl_2_, and CaCl_2_) used during impregnation lower the yield of levoglucosan, whereas Ni(NO_3_)_2_ increases it. 

Apart from a decrease in the yield of levoglucosan, Shimada et al. [104] reported a decrease in the yield of 5-hydroxymethyl furfural and methanol. Khelfa et al. [98] compared the results obtained by the impregnation of biomass components with MgCl_2_ and NiCl_2_ solutions. They determined that the presence of MgCl_2_ causes a substantial increase in the content of furan and pyran compounds originating from the decomposition of xylan. In turn, Fan et al. [100] covered cellulose, xylan, and lignin with KCl. The analysis of the distribution of the products conducted by Fan et al. [100] demonstrated that K facilitates the breaking of glycosidic bonds and the decomposition of glucose units and causes a substantial decrease in the yield of carbohydrates along with an increase in the yield of furans, aldehydes, and ketones. 

Chen et al. [105] reported that the pretreatment of rice straw with water, a dilute solution of hydrochloric acid, and a *bio*-*oil* aqueous *phase* resulted in the removal of AAEM present in biomass. As a result, the relative content of acids, ketones, furans, and phenols decreased in tar. However, the removal of AAEM increased the relative content of anhydrosugars (mainly levoglucosan) and facilitated the release of volatile compounds. 

Shen et al. [93] reported that the pyrolysis of rice husks in the presence of AEM lowered the content of acids and increased that of hydrocarbons in tar. The role of Mg in the form of MgO and MgCO_3_ in the yield of hydrocarbons present in tar is much more important than that of Ca (i.e., CaO, CaCO_3_). According to Hu et al. [94], the presence of AAEM promoted the decomposition and the reactions of decarboxylation/decarbonylation of thermally unstable heteroaromatic compounds which led to a decrease in tar yield and an increase in char yield. Xia et al. [97] suggested that the presence of Fe in tar facilitates the formation of ketones and acids in the temperature range of 400–600 °C. The char obtained in the presence of Fe at the temperature of 700–800 °C had a greater pore volume, a larger specific surface, and a higher graphitization degree of char. 

Collard et al. [96] ascertained that the impregnation of cellulose with Fe(NO_3_)_3_ and Ni(NO_3_)_2_ facilitates the re-ordering of aromatic rings which results in a decrease in the concentration of aromatic compounds in tar.

**Figure 8:**
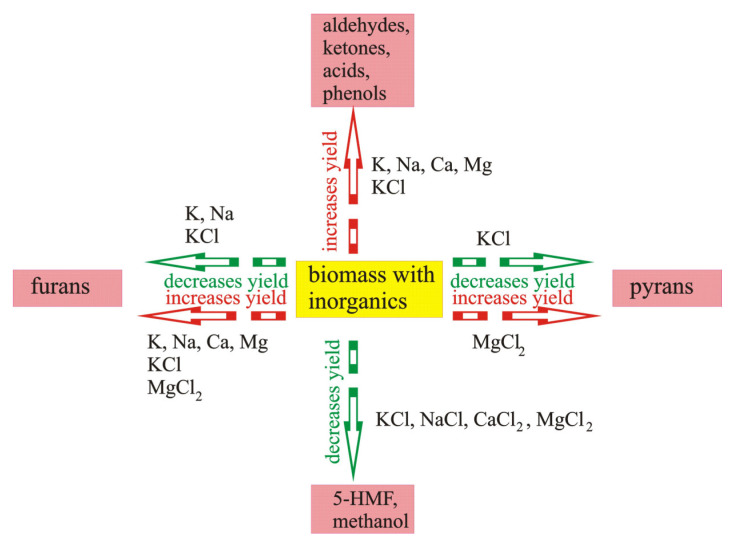
The influence of inorganic components on the yield of selected compounds: increase in yield of aldehydes, ketones, acids, and phenols influenced by K, Na, Ca, and Mg (data from [105]); KCl (data from [100]); decrease in yield of pyrans influenced by KCl (data from [91]); increase in yield of pyrans influenced by MgCl_2_ (data from [98]); decrease in yield of 5-HMF and methanol influenced by KCl, NaCl, CaCl_2_, MgCl_2_ (data from [104]); decrease in yield of furans influenced by K, Na (data from [49]); KCl (data from [91]); increase in yield of furans influenced by K, Na, Ca, and Mg (data from [105]); KCl (data from [100]); MgCl_2_ (data from [98]).

An overall summary of the influence of inorganic components on the yield of selected compounds is presented in Figure 8. 

The abovementioned makes it possible to systemize the effect of the influence of particular inorganic components naturally occurring in biomass, or added during impregnation, on the yield of selected organic compounds. 

In the scientific literature, there are certain contradictions with regard to the yield of organic compounds formed during pyrolysis. Some scientists hold the view that AAEM elements naturally occurring in the biomass, as well as Mg and K introduced in the form of chlorides, increase the yield of furans [98,100,105]. However, others suggest that Na and K naturally occurring in the biomass and K introduced in the form of KCl decrease the yield of furans [49,91]. There is only a single report devoted to other compounds that were identified in volatile products. Opinions and suggestions in these reports should be confirmed by a range of additional investigations. Nevertheless, the results presented in Figure 8 imply that the use of inorganics makes it possible to modify the composition of the pyrolysis products formed. 

**Figure 9:**
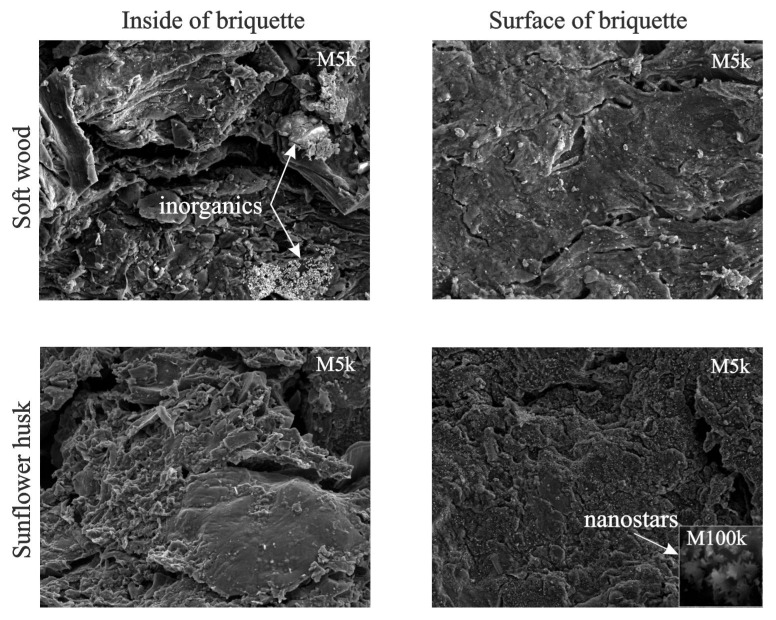
SEM images of pyrolyzed samples of soft wood and sunflower husks.

Figure 9 presents the SEM images of the inside and surface of the briquettes of pyrolyzed samples of soft wood and sunflower husk.

The visualization of these chars in the soft wood sample shows that inorganic components are located inside the briquette, whereas in the sunflower husk sample they are located on its surface. The location of inorganics inside the briquette in combination with a decrease in its porosity increases the yield of hydrocarbons in the composition of volatile products. Figure 1b and Figure 9 imply that the cumulation of inorganics on the surface of the char from sunflower husks can substantially change the nature of the interaction between the volatiles and char and decrease the contribution of hydrocarbons in the composition of volatile products [70]. 

This fact gives grounds to suggest that better effects of the influence of inorganic components on the interactions of volatiles and char with biomass can be obtained by covering the surface of a briquette with appropriately selected inorganic substances.

## 6. Conclusions

The results connected with the occurrence of interactions in blends of cellulose with lignin, lignin with hemicellulose, and hemicellulose with cellulose as well as those for the interactions between the products formed during pyrolysis were systemized. In addition, the effects of the influence of interactions between the biomass components on the formation of levoglucosan along with the yield and composition of pyrolysis products of lignocellulosic biomass were noted. Several conclusions were drawn from the discussion presented above:

(i) The occurrence/happening of interactions between all components is inevitable during the pyrolysis of a blend of single components or native biomass;

(ii) The interactions between cellulose and lignin influence the yield of char in a different way; an increase in the emission of volatile products and a decrease in the yield of tar were stated unequivocally; 

(iii) When considering the interactions between a blend of hemicellulose and cellulose, the yield of char and volatile products increases independently from the type of interacting component;

(iv) The presence of hemicellulose in studied samples decreases the yield of levoglucosan; 

(v) The inorganic components present in the biomass and added during impregnation, as well as the additions of organic compounds, cause changes in the yield and composition of pyrolysis products, including the yield of levoglucosan; 

(vi) The course of the pyrolysis process can be modified by the changes in the composition of the basic components and the location of inorganic components in pyrolyzed blends of various biomass types. 

The research connected with the explanation and verification of the effects of interactions taking place in the biomass during pyrolysis should be intensified in order to deepen the knowledge of the mechanism of the pyrolysis of lignocellulosic biomass and to increase the methods of processing biomass into alternative fuels, valuable chemicals for chemical syntheses, or other materials to be used in various industries. 

## Figures and Tables

**Table 1:** The effects of the interactions between cellulose and lignin occurring during pyrolysis.

Feedstock	Interacting Component	Main Effects	References
cellulose	lignin	increase in char yield	[41,48,57]
		lack of influence on char yield	[55,59]
		decrease in char yield	[52,61]
		increase in yield of volatile products	[52,57,60]
		decrease in tar yield	[41,56,57]
		increase in yield of tar insoluble in water decrease in yield of tar soluble in water	[52]
			[52]
lignin	cellulose	decrease in char yield	[58]
		decrease in yield of volatile products increase in yield of tar insoluble in water decrease in yield of tar soluble in water	[56]
			[56]
			[56]

**Table 2:** The effects of the interactions between hemicellulose and cellulose occurring during pyrolysis.

Feedstock	Interacting Component	Main Effects	References
hemicellulose	cellulose	increase in char yield	[43]
xylan	cellulose	increase in yield of volatile products	[51]
cellulose	xylan	increase in char yield	[65]
		increase in yield of volatile products	[44,56,66]
		decrease in tar yield	[44,65]
		increase in yield of tar soluble in water originating from cellulose	[56]

**Table 3:** The effects of the influence of the interactions between the biomass components on the yield of levoglucosan.

Interaction	Yield of Levoglucosan	References
cellulose–hemicellulose–lignin	decrease	[38,56]
cellulose–lignin	decrease	[43,53,55,72,87]
cellulose–lignin	increase	[42,44,52,59]
cellulose–hemicellulose	decrease	[43,44,51,52]

## Data Availability

Not applicable.
